# Deciphering host-parasitoid interactions and parasitism rates of crop pests using DNA metabarcoding

**DOI:** 10.1038/s41598-019-40243-z

**Published:** 2019-03-06

**Authors:** Ahmadou Sow, Thierry Brévault, Laure Benoit, Marie-Pierre Chapuis, Maxime Galan, Armelle Coeur d’acier, Gérard Delvare, Mbacké Sembène, Julien Haran

**Affiliations:** 1 Département de Biologie Animale, FST-UCAD, Dakar, Senegal; 2 BIOPASS, CIRAD-IRD-ISRA-UCAD, Dakar, Senegal; 3 CIRAD, UPR AIDA, F-34398 Montpellier, France; 4 AIDA, University Montpellier, CIRAD, Montpellier, France; 5 CIRAD, CBGP, Montpellier, France; 6 CBGP, CIRAD, INRA, IRD, Montpellier SupAgro, University Montpellier, Montpellier, France; 7 CBGP, INRA, CIRAD, IRD, Montpellier SupAgro, University Montpellier, Montpellier, France

## Abstract

An accurate estimation of parasitism rates and diversity of parasitoids of crop insect pests is a prerequisite for exploring processes leading to efficient natural biocontrol. Traditional methods such as rearing have been often limited by taxonomic identification, insect mortality and intensive work, but the advent of high-throughput sequencing (HTS) techniques, such as DNA metabarcoding, is increasingly seen as a reliable and powerful alternative approach. Little has been done to explore the benefits of such an approach for estimating parasitism rates and parasitoid diversity in an agricultural context. In this study, we compared the composition of parasitoid species and parasitism rates between rearing and DNA metabarcoding of host eggs and larvae of the millet head miner, *Heliocheilus albipunctella* De Joannis (Lepidoptera, Noctuidae), collected from millet fields in Senegal. We first assessed the detection threshold for the main ten endoparasitoids, by sequencing PCR products obtained from artificial dilution gradients of the parasitoid DNAs in the host moth. We then assessed the potential of DNA metabarcoding for diagnosing parasitism rates in samples collected from the field. Under controlled conditions, our results showed that relatively small quantities of parasitoid DNA (0.07 ng) were successfully detected within an eight-fold larger quantity of host DNA. Parasitoid diversity and parasitism rate estimates were always higher for DNA metabarcoding than for host rearing. Furthermore, metabarcoding detected multi-parasitism, cryptic parasitoid species and differences in parasitism rates between two different sampling sites. Metabarcoding shows promise for gaining a clearer understanding of the importance and complexity of host-parasitoid interactions in agro-ecosystems, with a view to improving pest biocontrol strategies.

## Introduction

Insect parasitoids are usually defined as species whose larvae develop by feeding in or on the body of an arthropod host, eventually killing it^1^. Insect parasitoids (herein, parasitoids) display great species diversity and a wide variety of biological and ecological traits. They can play an important role in the natural regulation of arthropod populations^2,3^. Approximately 10–25% of insects are thought to be parasitoids^1,4^ with as many as 2 million species worldwide^5^.

Biological control of agricultural insect pests using natural enemies, such as parasitoids, is a successful way of reducing the crop yield losses caused by these pests^6–9^ and is an ecologically-based alternative to insecticide use^10^. Such an approach includes the introduction of ‘exotic’ parasitoids (classical biological control) into new environments. Alternatively, native parasitoids can be mass-reared and periodically released in the field (augmentative biological control), when population sizes are not sufficient to provide effective control^11^. An abundance of native parasitoids can also be promoted through adequate manipulation of their habitats (i.e. conservation biological control), including manipulation of the crop’s microclimate, conservation of overwintering refuges, provision of alternative hosts and accessibility to essential food resources, such as flowers for adult parasitoids^12^.

A fast and reliable diagnostic of parasitism in target pests is critical for assessing the efficacy of biological control programs, but also for ecological studies dealing with host-parasitoid interactions and trophic webs^13,14^. Traditional methods used to identify parasitoids and assess parasitism rates in a pest population rely on rearing the host up to the emergence of adult parasitoids, or the dissection of host material. However, results from host rearing can often be biased by the differential mortality of hosts due to diseases or other common stresses occurring during the rearing process. In addition, identifying parasitoid species obtained by host rearing calls for the expertise of a taxonomist, which often reaches its limits for the identification of morphologically cryptic species. For the two conventional methods (rearing or dissection), the workload limits the number of samples that can be analyzed. Consequently, these methods are difficult to scale up to large sampling designs^15^. Molecular methods have opened up new prospects for identifying parasitoids and evaluating host-parasitoid interactions, early in the host egg stage, taking a Restriction Fragment Length Polymorphism approach (PCR-RFLP, see Papura *et al*.^16^), or in the larval stage using molecular analysis of parasitoid linkages (MAPL)^17^. MAPL is based on detecting either host DNA in adult parasitoids (MAPL-AP), or parasitoid DNA in tissues of host larvae (MAPL-HL) using species-specific PCR primers of target taxa^18^. The two methods have been shown to detect higher parasitism rates than traditional insect rearing methods^18^, but they were only applied using Sanger sequencing^13,19^, often limited by PCR success and sampling size. In a current context of increasing use of DNA metabarcoding via high-throughput sequencing (HTS), there is a need to operate a transition toward more efficient versions of these approaches. DNA metabarcoding is efficiently used for the detection of host-parasite interactions, as illustrated in a wide range of organisms^20,21,22^, including insects^23,24^. This approach provides reliable, rapid and accessible identification of both immature and adult specimens, even for non-taxonomists, and without *a priori* knowledge on the specific composition of the food web^25^. In addition, DNA metabarcoding can provide information that is not easily obtained through conventional host rearing and conventional DNA barcoding, such as multiparasitism^26^, since it consists in co-amplifying and co-sequencing several taxa in a single PCR reaction^27^. Lastly, DNA metabarcoding can be used to sequence a large number of samples at limited cost^28,29,30^, opening up the possibility of undertaking large-scale analyses. However, DNA metabarcoding may be subject to technical bias, which needs to be properly taken in account when studying host-parasitoid interactions^31^, such as potential DNA contamination (in the laboratory), or non-detection related to PCR biases^29^. The latter can be due to mismatches between primers and particular DNA targets, and to competition for co-amplification of a small proportion of parasitoid DNA in a large quantity of host DNA. Although adequate technical practice makes it possible to reduce biases inducing false positive or negative results in metabarcoding experiments^31,32,33^, an evaluation of the efficiency of DNA metabarcoding is lacking in systems other than soils^34^, and marine and freshwater environments^35,36,37^. Yet, it is crucial to evaluate detection sensitivity for an accurate and reliable diagnostic of pest parasitism in agro-ecosystems.

The aim of this study was to assess the relevance of DNA metabarcoding in identifying parasitoid species and their relative contribution to the natural control of a crop pest. As a study system, we used the millet head miner (MHM), *Heliocheilus albipunctella* (de Joannis) (Lepidoptera: Noctuidae), a key pest of pearl millet in sub-Saharan Africa. Crop losses due to this pest are greatly mitigated by natural enemies, including predators and parasitoids^38–40,41^, making it a relevant model for exploring the functional biodiversity involved in trophic webs and biological control. The relevance of this model is also supported by the fact that assemblages of parasitoids associated with MHM have been morphologically and genetically documented^41^, and that significant larval mortality generally occurs during rearing processes (e.g. ~47% larval mortality in Sow *et al*.^42^), which is a major obstacle for the assessment of parasitism rates.

In this study, we first assessed the detection sensitivity of the DNA metabarcoding protocol for reconstructing the MAPL of MHM, by testing the amplification of an artificial mix of DNA from hosts and parasitoids. Then, the parasitism rate was estimated by concurrently applying the DNA metabarcoding and rearing methods to MHM eggs and larvae collected in the field from two contrasting agricultural landscapes.

## Results

### Sequencing results and data filtering

We first conducted an Illumina MiSeq run to sequence 114 dilution test samples. A total of 929,364 reads (mean = 8,152, SD = 2,540) were generated, and after filtering, 928,344 reads (mean = 8,143, SD = 2,542) remained (see Dataset S1 and S2 for further details on raw and filtered sequencing data). We then conducted another Illumina MiSeq run to sequence 1,113 moth samples collected in the field. A total of 8,045,424 reads (mean = 6,749.5, SD = 2,467), with 87.5% of reads belonging to MHM (1 genetic cluster), 6.1% of reads belonging to *Masalia nubila* (2 genetic haplotypes-it was the eggs of this pest that were collected on the millet heads, and it was impossible to distinguish between them and those of MHM; these sequences were removed from the dataset after filtering), 2.1% of reads belonging to parasitoids (4 genetic haplotypes) and 4.2% of artefactual reads (chimeric sequences, contaminations, or reads from other taxa involved in the trophic web of MHM)(see Dataset S3 for further details on taxonomic abundances). After filtering according to the recommendation made by Galan *et al*.^43^, 7,404,164 reads were obtained (mean = 6,652, SD = 1,329)(see Dataset S4 for further details on filtered abundance data).

### Evaluation of the DNA metabarcoding approach for parasitoid detection

The metabarcoding approach was calibrated in two stages, (1) we first assessed the detection sensitivity of the DNA metabarcoding protocol by testing the amplification of artificial dilution gradients of the parasitoid DNAs in the host moth, and (2) we then evaluated detection variability between different stages of host development by sequencing samples collected in the field.

#### Detection of parasitoids from controlled host-parasitoid DNA dilutions

**Figure 1:**
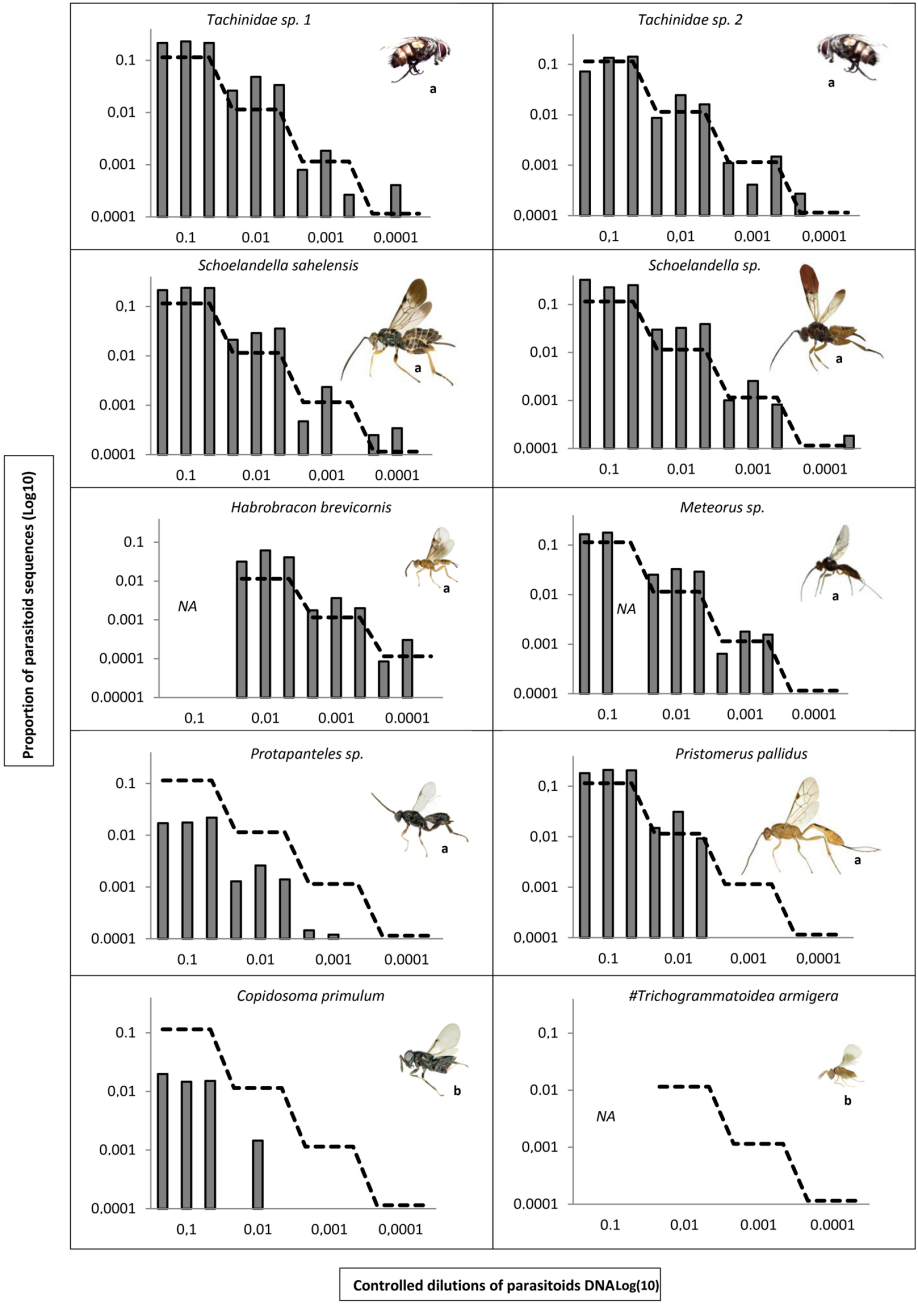
Proportion of parasitoid sequences observed from metabarcoding (y-axis) of controlled dilutions of parasitoid DNA in host DNA (x-axis) for each of the 10 major parasitoid species. For each parasitoid species, we mixed 7, 0.7, 0.07, or 0.007 ng of its DNA with 56 ng of DNA from the host, *Heliocheilus albipunctella*. The dashed line represents the expectation. Axes are in a log-scale. The three bars per dilution account for the three technical PCR replicates. NA: data is not available because of a lack of DNA availability for the parasitoid. #: a critical mismatch (in bold) was found between the parasitoid voucher sequence (5′-CATGCTTTT**T**TAATAATTTTTTTTTTTGT-3′) and the 3′ side of the reverse primer (MG-R: 5′-ACTATAAAARAAAYTATDA**Y**AAADGCRTG-3′). The scale of parasitoid images is **(a)** 300 µm for a length of 0.5 cm, **(b)** 200 µm for a length of 0.2 cm.

**Figure 2:**
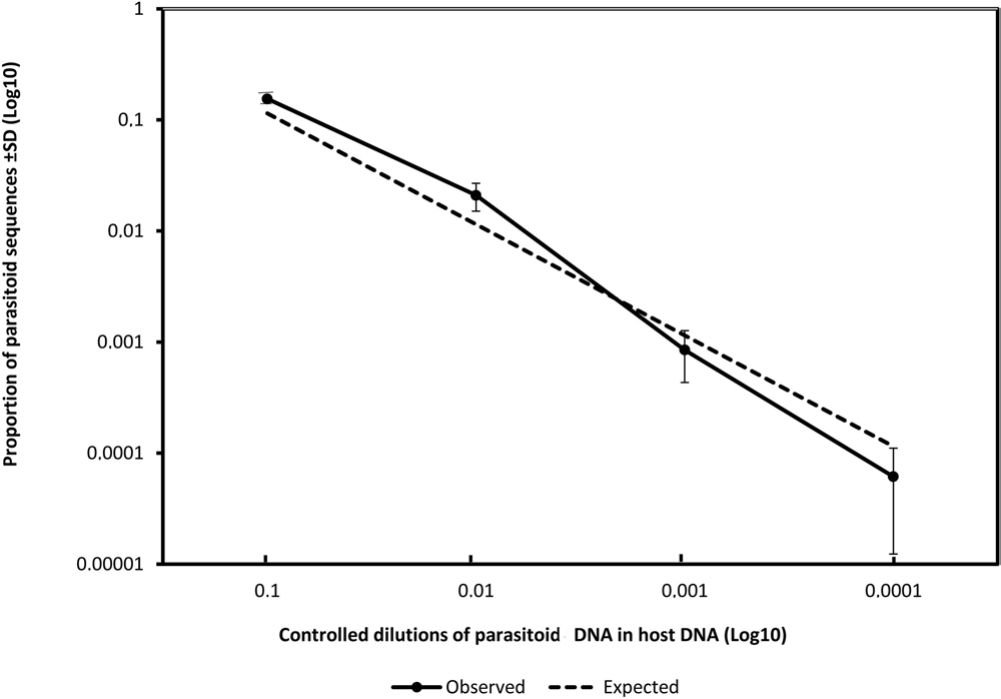
Proportion of parasitoid sequences observed from metabarcoding (y-axis) of controlled dilutions of parasitoid DNA in host DNA (x-axis) averaged over the 10 major parasitoid species. For each parasitoid species, we mixed 7, 0.7, 0.07, or 0.007 ng of its DNA with 56 ng of DNA from the host, *Heliocheilus albipunctella*. Bars represent the standard deviation. The dashed line represents the expectation. Axes are in a log-scale.

We evaluated the detection rate for DNAs from 10 parasitoid taxa mixed at different concentrations in host DNA (Figs 1 and 2). All the parasitoid taxa were detected except *Trichogrammatoidea armigera* (the main parasitoid of MHM eggs) (Fig. 1). We discovered a critical mismatch between the voucher sequence of this parasitoid species and the 3′ side of the reverse primer. Such a downward bias of the final proportion of the target species had already been observed in the metabarcoding of arthropod mock communities^43,44^.

Overall, there was a strong concordance between the proportion of parasitoid sequences observed and the abundance of parasitoid DNA relative to the quantity of host DNA (Fig. 2). The number of sequences obtained among technical replicates was remarkably stable. When considering as positive those samples with at least two of the three technical replicates that were positive, the detection threshold was 0.07 ng of parasitoid DNA for 56 ng of host DNA (i.e. a relative DNA abundance of ~0.001) for seven out of the nine parasitoid species (i.e. *Habrobracon hebetor*, *Schoelandella* sp., *Meteorus* sp., *Schoelandella sahelensis*, *Protapanteles* and the two species of tachinids; Fig. 1). The exceptions were *Pristomerus pallidus* and *Copidosoma primulum*, with a detection threshold of 0.7 ng and 7 ng of parasitoid DNA for 56 ng of host DNA, respectively (i.e. a relative DNA abundance of ~0.01 and ~0.1, respectively). At the highest relative abundances (0.1 and 0.01), the proportions of sequences for parasitoids were greater than expected, except for *C. primulum* and *Protapanteles* sp. (Figs 1 and 2). At the detection threshold for most species (0.001), parasitoid DNA was slightly under-represented, whereas host DNA was probably over-amplified by PCR competition effects (Fig. 2).

#### Detection of parasitoids from egg and larval hosts collected in the field

**Figure 3:**
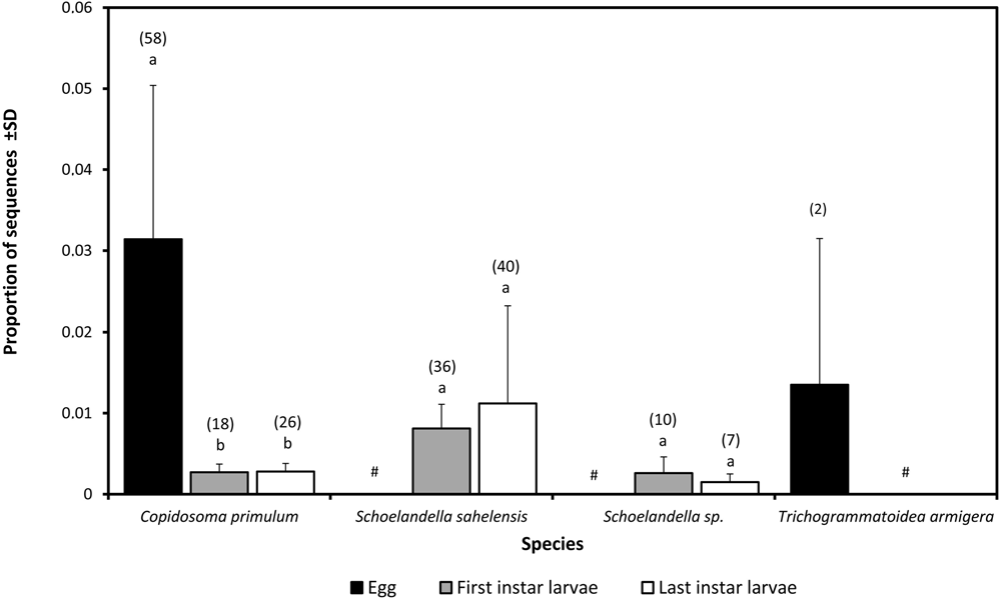
Proportion of sequences of four parasitoid species observed from DNA metabarcoding from 197 individual field samples of the millet head miner *Heliocheilus albipunctella* at different stages of development (eggs, early instar larvae, late instar larvae). *Trichogrammatoidea armigera* is an egg parasitoid, *Copidosoma primulum* is an ovo-larval parasitoid, *Schoelandella sahelensis* and *Schoelandella* sp. are larval parasitoids. Bars represent the standard deviation. Values above each bar represent the number of positive samples for each stage of the four parasitoid species. #: No data.

We evaluated the proportion of sequences of parasitoid species observed through DNA metabarcoding of individual moth samples collected in the field (*n* = 400) at different developmental stages (eggs, early instar larvae, late instar larvae). Four parasitoid species, namely *T. armigera* (Trichogrammatoidae), *C. primulum* (Encyrtidae), and the two cryptic species, *S. sahelensis* and *Schoelandella* sp. (Braconidae), were identified with 100% homology to our reference sequence database. The database consisted of 234 sequences of 658 bp of the *mitochondrial cytochrome oxidase 1* from species involved in the trophic web of MHM in the Senegalese agricultural system (GenBank accession numbers MF673564-MF673719, Table S1). The proportion of sequences of *T. armigera* in parasitized eggs was 0.0136 ± 0.0187 (*n* = 2). The proportion of sequences of *C. primulum* was higher in parasitized eggs (0.032 ± 0.020, *n* = 58) than in parasitized early-instar larvae (0.003 ± 0.002, *n* = 18) and late-instar larvae (0.003 ± 0.002, *n* = 26) (*t* = 2.8, *df* = 252.4, *P* = 0.006). The proportion of sequences of *S. sahelensis* was not significantly different between early-instar and late-instar larvae, with respectively 0.008 ± 0.003 (*n* = 36) and 0.011 ± 0.013 (*n* = 40) (*t* = −1.21, *df* = 159.41, *P* = 0.228). No difference was observed for parasitism between early- and late-instar larvae for *Schoelandella* sp., with respectively 0.003 ± 0.002 (*n* = 10) and 0.002 ± 0.001 (*n* = 7) (*t* = −0.41, *df* = 261.81, *P* = 0.680) (Fig. 3).

### Comparison of rearing and DNA metabarcoding for parasitoid detection

Following the evaluation steps, the detection of parasitism rates was compared between traditional rearing and DNA metabarcoding. The parasitism rates obtained with the two approaches were compared for overall parasitism estimates, but also for eggs and larvae parasitized by each species of parasitoid.

#### Overall parasitism rate

**Figure 4:**
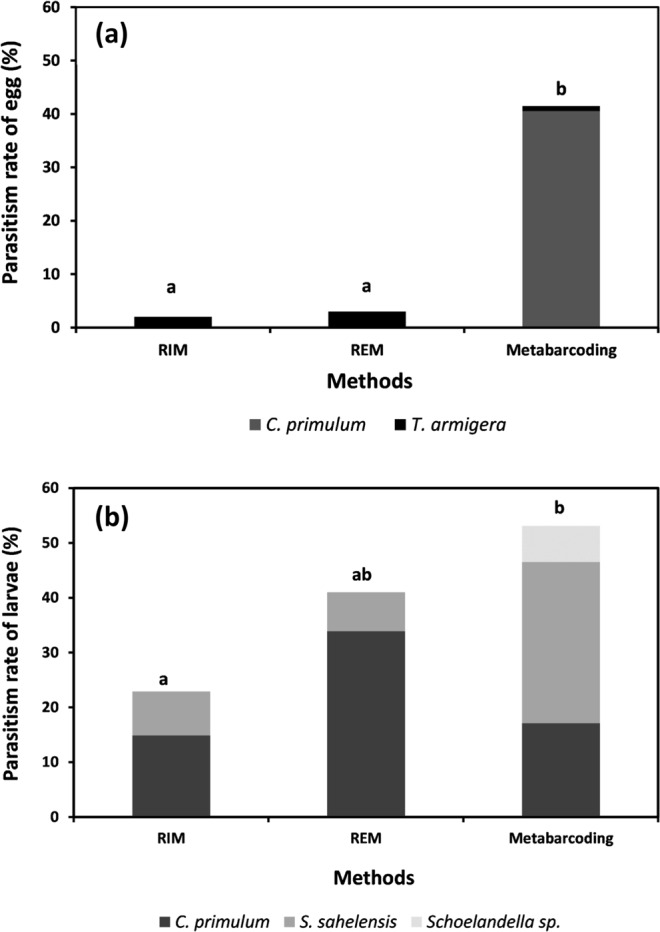
Comparison of DNA metabarcoding (*n* = 400) and rearing (*n* = 400) approaches in estimating the rate of parasitoid species from millet head miner *Heliocheilus albipunctella* eggs (**a**) and larvae (**b**) collected in the field. RIM (Rearing Including Mortality): the parasitism rate calculated including unhatched eggs or dead larvae. REM (Rearing Excluding Mortality): the parasitism rate calculated excluding unhatched eggs or dead larvae. Significant difference represented by the different letters (a and b).

**Figure 5:**
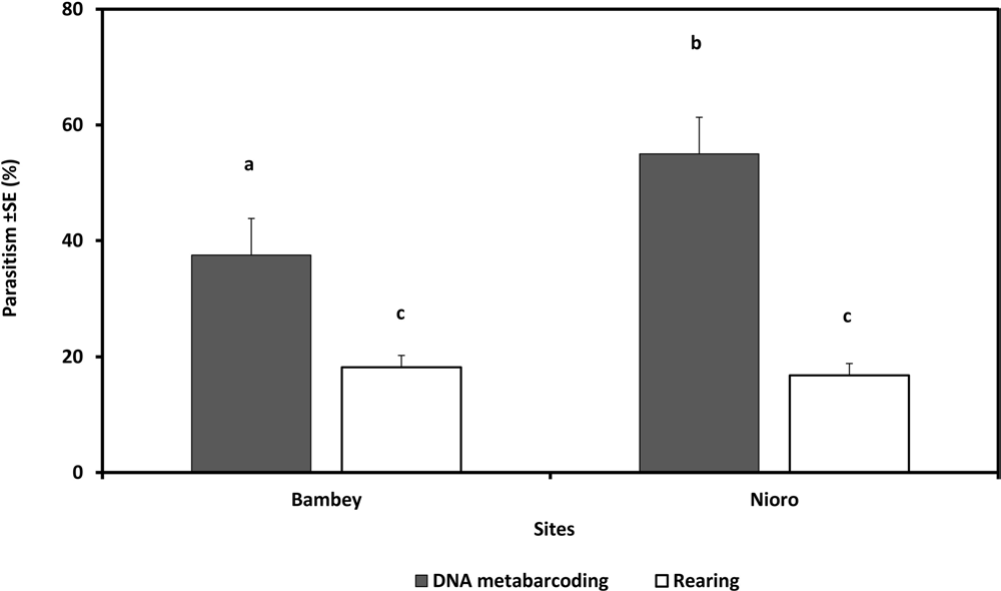
Overall parasitism rate of millet head miner *Heliocheilus albipunctella* parasitoids estimated by DNA metabarcoding and rearing methods according to the sample sites: Bambey and Nioro (Senegal). Significant difference represented by the different letters (a, b and c).

A high mortality rate was recorded for eggs (31.5% i.e. *n* = 190 on 400 moth samples, range 11.4–45.5) and larvae reared in the laboratory (56.3%, range 52.5–59.7). The egg parasitism rate obtained with rearing was significantly lower (3.0% with a range among fields of 2.0–3.0%, or 2.3% with a range of 0–3.3%, when taking mortality into account in the calculation or not, respectively) than that obtained by metabarcoding (42.0%, range 18.2–58.8%) (*F* = 68.8, *df* = 1, *P* < 0.001) (Fig. 4a). The larval parasitism rate obtained with rearing was also lower (22.1%, range 16.7–26.9%) than that obtained by metabarcoding (48.8%, range 40.7–56.1%) (*F* = 21.61, *df* = 1, *P* < 0.001), but there was no significant difference between metabarcoding and rearing when taking mortality into account in the calculation (39.3%, range 21.4–48.3%) (*F* = 2.99, *df* = 1, *P* = 0.085) (Fig. 4b). The parasitism rate estimated by metabarcoding was significantly higher in Nioro compared to Bambey, with respectively 55.0 and 37.5% (*F* = 12.65, *df* = 1, *P* < 0.001), whereas it was not significantly different between the two sites for the rearing methods (*F* = 0.019, *df* = 1, *P* = 0.89), with 18.2 and 16.8% in Bambey and Nioro, respectively (Fig. 5). Multiparasitism was observed on 1.4% (range, 0–6.0) of eggs and 7.9% (range, 0–16) of larvae analyzed by metabarcoding. No case of multiparasitism was observed for rearing.

#### Parasitism rate per parasitoid species

The mean proportions of eggs parasitized by *T. armigera*, a main MHM egg parasitoid, were low and not significantly different between the two methods (2.5% and 1.0%, respectively; *F* = 1.02, *df* = 1, *P* = 0.313) (Fig. 4a). However, for this species, the comparison between the two methods was challenged by the downward bias of detection for metabarcoding due to the 3′ mismatch with the reverse primer. The parasitoid *C. primulum* was a major parasitoid with 40.5% (range, 18.2–55.9) of parasitized eggs with metabarcoding and 17.1% (range, 11.9–22.7) of parasitized larvae with metabarcoding. The prevalence of *C. primulum* estimated by rearing was null in eggs and of 22.3% (range, 6.1–44.8) in larvae. When taking mortality into account in the calculation, the prevalence of *C. primulum* in larval hosts estimated by rearing became higher (33.9%, range, 14.3–44.8) than with metabarcoding (*F* = 39.2, *df* = 1, *P* < 0.001). The mean proportion of *S. sahelensis* was significantly higher with metabarcoding (29.4%) than with rearing, taking or not mortality into account in the calculation (7.1 and 8.0%, respectively) (*F* = 4.531, *df* = 2, *P* < 0.0111) (Fig. 4b).

## Discussion

To understand host-parasitoid interactions, it is necessary to estimate parasitism rates and identify parasitoid species accurately^24,45–47,48^. Molecular approaches can provide more information than conventional methods, for a better understanding of interactions between host or prey species and their natural enemies, including parasitoids^18,24^ and predators^49^. A better understanding of these interactions is a fundamental basis for successful biological control programs based on the introduction or enhancement of parasitoids^6,8,50^. This study showed that metabarcoding is a reliable support for assessing the specific and functional diversity of the parasitoids of insect pests in an agro-ecosystem, and that it performs better than rearing.

Recent studies showed the effectiveness of DNA metabarcoding for identifying individuals involved in host-parasitoid interactions at different trophic levels^23,41^, in comparison to standard DNA barcoding and morphological approaches^24^. However, little attention has been paid to the sensitivity (detection threshold) of this method, particularly regarding the importance of more effectively taking into account PCR biases for an accurate and reliable diagnostic of parasitism. Our results showed that the universal primers^43^ used for DNA metabarcoding were effective for the detection of parasitoid DNA, even when present in very small quantities in host tissues. Under controlled conditions, we were able to detect parasitoid DNA up to a dilution ratio of 0.001 for seven host-parasitoid species pairs. For the single exception (excluding *T. armigera* and *C. primulum* with a primer mismatch; see next paragraph below), the detection threshold was 0.01. Therefore, a dilution ratio of 0.01 enabled systematic detection of parasitoid DNA in our system provided a primer-target complementarity. This ratio was higher than the ratios of parasitoid/host reads encountered for field samples (mean = 0.006, range 0.001–0.01). Given the absence of major PCR bias (highlighted in the dilution tests), it is reasonable to assume that these ratios correspond to the ratio of parasitoid tissues in the host larva encountered in the field. Consequently, we argue that there is little risk of missing parasitoids in host eggs and larvae collected in the field. Liang *et al*.^51^ obtained similar results using qPCR, with an egg detection threshold of 0.25 ng for parasitoid DNA [(*Fopius arisanus* (Sonan)] in 40 ng of host DNA [*Bactrocera dorsalis* (Hendel)], for a dilution ratio of ~0.006. In addition, our detection threshold appeared sensitive enough to detect all the developmental stages of our parasitoids, including embryonic stages. This point is essential for estimating parasitism rates by DNA metabarcoding, because it suggests that even the earliest stages of parasitoids (which can be one egg in the host larva) can be effectively detected.

Metabarcoding recovered parasitoid tissues in the majority of host parasitoid pairs, but two cases were problematic, likely due to competition due to PCR bias or manipulation error. Competition related to PCR bias between Lepidoptera and Hymenoptera was not apparent, except for the egg parasitoid *T. armigera*, which presented a sequence mismatch with the EPT-MG-R primer (see^43^ and legend of Fig. 1). Interestingly, while *T. armigera* DNA was not detected in controlled mixtures with host DNA, this species was still detected at a low rate (1%) in eggs collected in the field (1%). Furthermore, in these detection cases, the host DNA was not amplified at all (i.e., zero sequences). This indicates that only egg parasitoids that are at very advanced stages of development (i.e., late instars) can be detected, which means that the positive samples observed in metabarcoding are probably those whose parasitoid tissue was very abundant in the host and false negatives are expected for samples with parasitoid tissue in low abundance in the host. This limit of our method resulted in an under-estimation of the parasitism rate of egg parasitoids. However, it can be easily lifted using one base degeneracy for the problematic position of the reverse primer. The high detection threshold for *C. primulum* (0.1) may also be partly related to imperfect primers, with a 5′ mismatch detected in the reverse primer, which is yet expected to reduce amplification efficiency much less seriously than a 3′ mismatch.

The metabarcoding molecular approach is an increasingly promising alternative to conventional methods in parasitism diagnostics. In our case study of a complex of ten parasitoid species recorded from MHM, the three main key parasitoid species were detected by metabarcoding and rearing (*T. armigera*, *C. primulum*, *S. sahelensis*). In addition to these, the metabarcoding has also detected *Schoelandella* sp. a cryptic species of *S. sahelensis*. These species are key parasitoids of MHM, i.e., dominant and ubiquitous species inventoried by Sow *et al*.^41^. Other species are rare or very rare and are usually not detected in surveys conducted on a reduced number of sites and samples.

DNA metabarcoding resulted in significantly higher estimates of parasitism rates than rearing, at both sampling sites, when excluding mortality from the calculation. No significant difference between DNA metabarcoding and rearing was observed when mortality was included. This information is interesting because it suggests that much of the mortality of host larvae is apparently related to parasitism. In host eggs, we detected two egg parasitoid species, *C. primulum*, and *T. armigera* with DNA metabarcoding, whereas only *T. armigera* was obtained with rearing. This was predictable because *C. primulum*, which is an ovo-larval parasitoid, parasitizes its host at the egg stage, but adult parasitoids emerge in late-stage larvae. However, even though this parasitoid largely dominates egg parasitism in DNA metabarcoding, it does not have a direct impact on egg mortality. The mismatches detected between the voucher sequence of *T. armigera* and the 3′ side of the reverse primer sequence prevented us from making a comparison between DNA metabarcoding and rearing for this species. Nevertheless, the parasitism rate due to this species is globally low (<4%). The larval parasitism rate obtained with rearing may have been underestimated due to host larva and parasitoid mortality (which reached a high level in our case). Host larva mortality remains the main limitation for an accurate and reproducible estimation of parasitism rates in the rearing laboratory^52,53^. In this case, DNA metabarcoding is useful for estimating parasitism rates because it can be used to detect parasitoids in the host body, whether the larval parasitoid is alive or dead^51^. For example, we can take the case of *S. sahelensis*, which was detected by both methods (DNA metabarcoding and rearing), and its detection threshold (~0.001) was low enough to detect all parasitoid stages. With DNA metabarcoding, the proportion of larval hosts parasitized by this species was quadrupled compared to rearing. In addition, this parasitoid largely dominated larval parasitism compared to *C. primulum* with DNA metabarcoding. In contrast, with rearing, the proportion of larval hosts parasitized by *C. primulum* was greater than for *S. sahelensis*. Consequently, DNA metabarcoding is very effective in ‘quantitatively’ estimating the impact of each parasitoid species involved in the natural regulation of the pest. However, it does not predict the survival of parasitoids as a result of host defense mechanisms.

Our results also showed that the parasitism rate estimated by DNA metabarcoding was significantly higher at Nioro than at Bambey, while no difference was observed between sites with rearing. Interestingly, Nioro has a typical savanna vegetation type displaying many spontaneous plants that could serve as reservoirs of alternative lepidopteran hosts. This could explain the higher density of parasitoids observed in this area. We thus showed that DNA metabarcoding can be more accurate and reliable for comparing parasitism between different agro-ecological contexts.

In this study, metabarcoding also enabled us to detect two cryptic species, or species in the process of genetic speciation, *S. sahelensis* and *Schoelandella* sp., and to measure their respective frequency. *S. sahelensis* played a greater role than *Schoelandella* sp. in MHM parasitism, and the proportion of sequences for *S. sahelensis* was much greater on older larval stages compared to *Schoelandella* sp. This suggests that *S. sahelensis* larvae grow faster than *Schoelandella* sp. larvae, and therefore kill the host larva earlier. In addition, we showed in a previous study that the species complex within *Schoelanedella* spp. also parasitized *Masalia nubila* and *Helicoverpa armigera*, two other lepidopteran pests observed on millet^41^. Considering that *Schoelandella* sp. is less abundant on MHM compared to *S. sahelensis*, we suggest that *Schoelandella* sp. is a potential generalist (several hosts) and uses MHM as an alternative host. Complexes of cryptic species are generally ignored in rearing experiments^54^, yet they are widely encountered in parasitic Hymenoptera^55^.

Competition between species within a common host is a major driver of the survival of a parasitoid species^56–59^. Interspecific competition for host resources can be detrimental to achieving biological control of a pest species, by affecting the establishment or efficacy of parasitoid species^60^. This study highlighted several cases of multi-parasitism and provided information on the existence of competition for resources between parasitoid species. Competition was observed at interspecific level for several pairs of species: *C. primulum* vs. *T. armigera* in eggs (frequency = 0.009) and *C. primulum* vs. two *Schoelandella* cryptic species in larvae (frequency = 0.05). Interspecific competition between *C. primulum* and *T. armigera* may partly explain the low levels of *T. armigera* parasitism observed with rearing. In the same host individual, larvae of *C. primulum* might develop defense mechanisms against *T. armigera* larvae. Strand *et al*.^61^ showed that in the case of multiparasitism between the polyembryonic parasitoid *Copidosoma floridanum* Ashmead (Encyrtidae) and the solitary endoparasitoid *Microplitis demolitor* Wilkinson (Braconidae) in the host *Pseudoplusia includens* Walker (Noctuidae), most multiparasitized hosts produced *C. floridanum* adults or died without any parasitoid emergence. This may also partly explain the high rate of larval mortality obtained with rearing, which was probably increased by competition linked to multiparasitism between our different parasitoid species. In the future, it would be interesting and original to metabarcode dead individuals during rearing to check, (1) if they were parasitized and (2) if so, which species of parasitoid caused most mortality.

While metabarcoding enabled a better description of the assemblages and abundance of parasitoids in host larvae, this approach still suffers from some limitations in deciphering the details of relationships between parasitoids. For instance, it cannot be used to predict the outcome of competition between parasitoid species: whether the species neutralize each other, or whether a given species has a greater tendency to take advantage of this competition^57^. Indeed, detection of a host does not necessarily indicate survival up to the adult stage^62^.

Information on biological traits, such as the solitary or gregarious behavior of parasitoid species (intraspecific competition), is also difficult to obtain because the amplified sequences of parasitoids cannot provide an evaluation of the number of specimens parasitizing a host larva. In addition, in DNA metabarcoding, the simultaneous detection of different sequences of parasitoids does not provide information on the nature of competition between parasitoid species, whether it is hyperparasitism or multiparasitism, without knowing the biology of the species. For all these points, estimation of the biological traits of parasitoids via DNA metabarcoding will benefit from the parallel use of conventional methods, such as the rearing and dissection of host larva.

In conclusion, our results showed that DNA metabarcoding can considerably improve our understanding of the host-parasitoid interactions and parasitism rates of crop pests. This approach is very sensitive, since the DNA of parasitoids can be successfully detected even when mixed in very small quantities with the host DNA. While an improvement in universal primers is still necessary to improve the recovery of parasitoid species, this study showed that metabarcoding makes it possible to (i) assess more precisely and reliably the level of parasitism of a crop pest among contrasting agro-ecological contexts, including the specific richness and diversity of the parasitoid community, and to (ii) more effectively assess multiparasitism and cryptic species, which are not necessarily detected through traditional rearing methods. This molecular approach should be combined with rearing methods to gain a better understanding of host-parasitoid interactions (especially competition) and bio-ecological traits of parasitoid species, and to identify the most effective species for further development of classical or conservation biological control programs.

## Methods

### Insect sampling

Millet head miner (MHM) eggs and larvae were collected in the field in the 2016 cropping season in four millet fields located near Bambey (14°43′0.79″N; 16°30′5.56″O) and Nioro du Rip (13°45′20.39″N; 15°47′12.29″O), in the ‘Peanut Basin’, the main area of millet production in Senegal. Bambey has a Sahelian-Sudanese climate, with a short rainy season (400–500 mm) from July to October (monsoon). The landscape is savannah with parklands (mainly *Faidherbia albida*, *Balanites aegyptiaca*, *Adansonia digitata*) and shrubs (mainly *Guiera senegalensis*) distributed across the agricultural matrix. Nioro du Rip has a Sudano-Sahelian climate with more rain (700–800 mm). The landscape is composed of savannah with shrubs (mainly *Piliostigma reticulatum*) and a few trees (mainly *Cordyla pinnata* and *Anogeissus leiocarpus*), and of semi-natural areas including rangelands and woodlands. Sampling was conducted from early to late September 2016 in each selected millet field, at three distinct phenological stages corresponding to the successive development of immature stages of MHM: panicle emergence corresponding to egg laying by MHM females, panicle with grains at the milk stage for 1^st^ and 2^nd^ instar larvae, and panicle with grains at the dough stage for 3^rd^ and 4^th^ instar larvae. In all, 800 specimens were collected, 200 from each millet field [Bambey 1 (66 eggs and 134 larvae), Bambey 2 (82 eggs and 118 larvae), Nioro 1 (70 eggs and 130 larvae), Nioro 2 (68 eggs and 132 larvae)]. Half of the sample was randomly selected for rearing in the laboratory, while the other half was kept in 90% ethanol for molecular analyses. Therefore, a total of 400 specimens (143 eggs and 257 larvae) were analyzed for host rearing and for DNA metabarcoding, respectively.

### Assessment of parasitism with host rearing

Eggs and larvae collected in the field were monitored in the laboratory for parasitism diagnostics. Eggs were sorted and incubated at room temperature in small pillboxes (10–25 eggs per box) until hatching or the emergence of parasitoids. Larvae were individually incubated at room temperature in 12-well culture plates (Fisher Scientific, France) filled with an artificial diet (Southland Products, USA), until pupation or the emergence of parasitoids or hyperparasitoids. Parasitoids emerging from eggs and larvae were preserved in 90% ethanol and identified by GD, using taxonomic keys^63–66^, or by comparing specimens with reference collections held at the British Museum (London, UK) or at the CBGP (Montpellier, France). The parasitism rate was calculated as the ratio of the number of parasitized eggs or larvae to (1) the total number of eggs or larvae, or (2) the number of hatched eggs or live larvae, i.e. with or without taking mortality into account, respectively.

### Assessment of parasitism with DNA metabarcoding

In order to verify the reliability and sensitivity of DNA metabarcoding, we first estimated (1) its ability to detect parasitoids in host tissues despite the imbalance in DNA ratio, (2) its sensitivity to several host-parasitoid assemblages and (3) its reproducibility among technical replicates. DNA metabarcoding was then applied to all larvae from the millet fields in order to estimate the parasitism rate.

### DNA extraction and dilution

DNA was extracted using the EZ-10 96-well plate DNA Kit for Animal Samples (Biobasic Inc., Canada). Small size specimens, such as micro-Hymenoptera and MHM eggs were extracted using the tube version of this kit (i.e. EZ-10 Spin Column Animal DNA Mini-Preps Kit, Biobasic Inc., Canada) to increase the yield of genomic DNA. All extractions were carried out according to the manufacturer’s protocol and by non-destructive lysis, corresponding to individual incubation overnight in a mixture of 300 µL of Animal Cell Lysis Solution and 20 µL of Proteinase K at 55 °C in an oven, with stirring.

In order to test sensitivity under controlled conditions, we extracted a single adult of the 10 major MHM parasitoid species^41^ (Table S1). The DNA concentration for each specimen was measured using a Qubit™ dsDNA HS Assay Kit (Qubit® 2.0 fluorimeter, Thermofisher, Canada), according to the manufacturer’s protocol. We also mixed eight MHM laboratory larvae known to be free of parasitoids for a single host DNA extraction. We then carried out serial dilutions on DNA extracts of known concentrations of parasitoids in proportions of 1:8, 1:80, 1:800, and 1:80000, relative to the initial concentration of the host DNA. These DNA mixtures were brought to a final volume of 2 µL, containing 1 µL of host DNA (56 ng/µL) and 1 µL of each dilution range (7, 0.7, 0.07, and 0.007 ng/µL) for each parasitoid species. Since we lacked DNA for the parasitoids *Habrobracon brevicornis* and *Trichogrammatoidea armigera*, we did not include the 1:8 dilution for these two species.

### PCR and Illumina sequencing

A 133 bp fragment of *cytochrome c oxidase I* (COI mini-barcode), was amplified from DNA samples using the primers and the two-step PCR protocol previously described in Galan *et al*.^43^, and sequenced on a MiSeq Illumina platform. This mini-barcode was found to discriminate parasitoid species of MHM and offered the advantage of amplifying degraded DNA, or rare DNA. Several negative controls (*n* = 10) were included, according to recommendations made by Galan *et al*.^33^. We performed 3 technical replicates on each DNA extract of mock and field samples in order to control the stochasticity of the PCRs and validate the positive results^67^.

A first amplification step on the short 133 bp COI fragment was carried out using universal primers (MG-LCO1490 5′-ATTCHACDAAYCAYAARGAYATYGG-3′ and MG-R 5′-ACTATAAAARAAAYTATDAYAAADGCRTG-3′) modified by Illumina 5′ end overhangs. The PCR reactions were carried out in a final volume of 10 µL containing 5 µL of Multiplex Master Mix (Qiagen, Hilden, Germany), 0.5 μM of each primer and 2 µL of DNA. The PCR conditions of this first step consisted of initial denaturation at 95 °C for 15 min, followed by 40 denaturation cycles at 94 °C for 30 s, hybridization at 45 °C for 45 s and extension at 72 °C for 30 s, and a final extension step at 72 °C for 10 min.

A second PCR step was performed to add individual-specific multiplexing tags (called index i5 and index i7), consisting of short 8 bp sample-specific sequences and the Illumina adapters (named P5 and P7), at the 5′ ends of each amplified DNA fragment, to the first PCR. Since all PCR products were mixed together (multiplexing) for MiSeq sequencing, indexes made it possible to identify the origin of the sequences and reassign them to each sample (demultiplexing). This second PCR was carried out in a total volume of 10 µL containing 5 µL of Multiplex kit (Qiagen, Germany), 0.7 μM of each primer, and 2 µL of products of the first PCR for each sample. PCR conditions consisted of an initial denaturation step at 95 °C for 15 min, followed by 8 denaturation cycles at 95 °C for 40 s, hybridization at 55 °C for 45 s and extension at 72 °C for 60 s, and a final extension step at 72 °C for 10 min.

The volume-to-volume mix of all the PCR products (4 µL per sample) was screened based on fragment length resulting from excision on 1.5% agarose gel. The pool of specific PCR products was cut to the expected size (328 bp corresponding to the size of the amplicon, including the gene-specific primers, sequencing primers, indexes and adaptors) under ultraviolet light. The resulting library was purified with the PCR purification kit (Macherey-Nagel, Germany), estimated by quantitative PCR (KAPA kit, Kapa Biosciences), then sequenced on a MiSeq sequencer with a 500v2 kit (Illumina, USA).

### Analyses of the MiSeq data set

Raw data from the Illumina MiSeq were deposited in the Dryad data repository (https://datadryad.org/) and available from this link: 10.5061/dryad.sj6mf40. We processed the paired-end sequencing data from the Illumina MiSeq system with the FROGS pipeline^68^ (https://github.com/geraldinepascal/FROGS.git). We preliminarily used a home-made script available at Dryad data repository (10.5061/dryad.sj6mf40) to merge pair sequences into contigs with FLASH v. 1.2.11^69^ and trim primers with cutadapt v. 1.9.1^70^. This script is particularly useful when using FROGS on data produced with primers that vary in size between reads, which is not covered by the pre-process step of FROGS. In our study, size variation in primers resulted from the addition of heterogeneity spacers in adapters during the library construction to shift the reading frame^71^. In FROGS, we then filtered sequences by length (expected value of 133b ± 10b), dereplicated sequences, removed chimeras using the algorithm of Edgar *et al*.^72^ implemented in VSEARCH v. 1.1.3 and clustered sequences with SWARM v. 1.3.0 using a local clustering threshold using the default d-value (d = 1)^73^.

We returned taxonomic affiliations for each OTU (Operational Taxonomic Unit) using a reference database previously constructed by Sow *et al*.^41^. The database consisted of 234 sequences of 658 bp of the mitochondrial *cytochrome oxidase 1* from species involved in the trophic web of MHM in the Senegalese agricultural system, and included 35 species, of which 13 were parasitoid species, 19 predator species and 3 herbivore species^41^ (GenBank accession numbers MF673564-MF673719, Table S1). Sequences absent from our database were compared by NCBI Blast + on public databases [BOLD Systems v. 3 (http://www.boldsystems.org), and GenBank (www.ncbi.nlm.nih.gov/genbank/)]. The identification was considered as ‘valid’ from a similarity threshold of 99 to 100%. Below this threshold, specimens were considered unidentified at species level.

Filtering for false positives was performed following the strategy proposed by Galan *et al*.^33^. In short, we discarded positive results associated with sequence counts below two OTU-specific thresholds, which checked respectively for cross-contamination between samples (using ten negative controls for extraction and PCR) and incorrect assignment due to the generation of mixed clusters on the flowcell during Illumina sequencing (using a global false index-pairing rate of 0.2%, based on estimates from Galan *et al*.^33^ Kircher *et al*.^74^). Finally, for each identified parasitoid, the number of sequences obtained in a PCR sample was converted into binary information (presence/absence) and DNA samples were considered positive if presence was confirmed in at least two of the three technical replicates^75^.

### Statistical analyses

A one-way Anova was used to test for a difference between DNA metabarcoding and rearing methods in estimates of the overall parasitism rate (including mortality, or not) and of the parasitism rates for each parasitoid species. A Wilcoxon paired test was used to test for a difference in the mean proportion of parasitoid sequences estimated by DNA metabarcoding between life stages of the host. Statistical analyses were performed with R software V. 3.2.3^76^.

## Supplementary information

Supplementary informations
