# Supplementary material for: Deciphering host-parasitoid interactions and parasitism rates of crop pests using DNA metabarcoding
Source: Sci Rep. 2019 Mar 6;9:3646. doi: 10.1038/s41598-019-40243-z (PMC6403368; doi:10.1038/s41598-019-40243-z)
Supplement: Supplementary file 1 — Supplementary informations [file 41598_2019_40243_MOESM1_ESM.pdf]

Deciphering host-parasitoid interactions and parasitism rates of crop pests using DNA metabarcoding.

Ahmadou Sow[a,b,]*, Thierry Brévault[b,c,d], Laure Benoit[e,f], Marie-Pierre Chapuis[e,f], Maxime Galan[g], Armelle Cœur d'Acier[g], Gérard Delvare[e,f], Mbacké Sembène[a,b], Julien Haran[e,f]

[a] Département de Biologie Animale, FST-UCAD, Senegal

[b] BIOPASS, CIRAD-IRD-ISRA-UCAD, Dakar, Senegal

[c] CIRAD, UPR AIDA, F-34398 Montpellier, France

[d] AIDA, Univ Montpellier, CIRAD, Montpellier, France

[e]CIRAD, CBGP, Montpellier, France

[f]CBGP, CIRAD, INRA, IRD, Montpellier SupAgro, Univ. Montpellier, Montpellier, France

[g]CBGP, INRA, CIRAD, IRD, Montpellier SupAgro, Univ. Montpellier, Montpellier, France

Supplementary information

Table S1. List of the main parasitoid species identified for the millet head miner *Heliocheilus albipunctella* in Sow et al. (2018) and used to assess the DNA metabarcoding detection threshold.

File S1. Bash script for read preparation before FROGS processing (preprocess_2steps_forFrogs_v0.2.sh).

File S2. and its parameter file (preprocess_2steps_forFrogs_parameters_file.txt) set for the minibarcode from Gillet et al. (2015).

Table S1. List of the main parasitoid species identified on the head miner *Heliocheilus albipunctella* and used for the DNA metabarcoding calibration. This list was established thanks to the study of Sow et al. 2018.

**Order**	**Species**	**Parasitized stage**	**GenBank accenssion numbers**
**Diptera**			
Tachinidae	*Tachinidae sp1* (Unidentified)	Larval parasitoid	MF673591
	Tachinidae sp2 (Unidentified)	Larval parasitoid	MF673593
**Hymenoptera**			
Braconidae	*Bracon brevicornis* (Wesmael, 1838)	Larval parasitoid	MF673597
	Meteorus sp	Larval parasitoid	MF673599
	Protapanteles sp	Larval parasitoid	MF673608
	Schoenlandella sahelensis (Huddleston & Walker, 1988)	Larval parasitoid	MF673609
	Schoenlandella sp	Larval parasitoid	MF673613
Encyrtidae	*Copidosoma primulum* (Mercet, 1921)	Egg parasitoid	MF673617
Ichneumonidae	*Pristomerus pallidus* (Kriechbaumer, 1884)	Larval parasitoid	MF673618
Trichogrammatidae	*Trichogrammatoïdea armigera* (Manjunath, 1972)	Eggs parasitoid	MF673622

File S1. Bash script for read preparation before FROGS processing

#!/bin/bash

on error exit flag
set -e

#debug flags
#set -xv

date : 20170214
version : 0.01
authors : MP Chapuis
licence : GPL
#
#########################

Illumina MiSeq Dataset analysis by FROGS

Short description:

From demultiplexed fastq.gz files produced from an Illumina MiSeq amplicon library, this script merges read 1 and read 2 (with flash, cuts contigs from both forward and reverse primers (with cutadapt) and collects processed fastq.gz files with a sufficient number of sequences in an archive.

USAGE: bash preprocess_2steps_forFrogs_v0.2.sh <parameter file>

Long Description:

This script is designed for two steps-PCR protocols. This script was originally devised for performing the primer removal and merging of reads instead of the pre-process step of FROGS that fails under certain circumstances. The final output gz.tar of this script can be uploaded in FROGS to be used in the pre-process step using Custom protocol (i.e. without PCR primers) for de-replicating sequences and filtering them on size and quality. This script is particularly useful when using FROGS on data produced with primers that vary in size between reads. For example, when spacers are added in adapters during the library construction to shift the reading frame.
Size variation in primers is not allowed in the pre-process step of FROGS. This script is also particularly useful when using FROGS on data of poor quality that affects primarily the end of reads that can be used in bioinformatics analyses.

In such a case, the merging of reads does not work with an overlap computed from expected read length and expected amplicon length.

Indeed, FROGS computes the overlap values (overlap in flash) from the information provided by the user on read size, expected amplicon size, minimum amplicon size and maximum amplicon size.

Since some of these parameters (minimum and maximum amplicon size) are also used for the filtering on size, a conflict appears in setting these parameters in FROGS (between success of merging and success of filtering).

The log files are gathered into a file named from the input parameter OUTPUT_FILE with the extension ".preprocess.log.tar".

The final files are gathered into a file named from the input parameter OUTPUT_FILE with the extension ".preprocess.gz.tar".

The script will work on all fastq.gz files located in the directory DIR.

Merging of reads 1 and 2 with flash: 2 input parameters controls for the min and max lengths of the overlap tolerated (MIN_FLASH and MAX_FLASH in min overlap and max overlap, respectively). Removal of primers with cut-adapt: 2 input parameters define the sequences of forward and reverse primers (PRIMER_F and PRIMER_R).

The parameter overlap is set as the length of the primer - 1 (as in FROGS). Note that by default the value of the overlap is 3. Note that it is still possible to use an overlap length between adapters and read shorter than the adapter length by modifying the values of the parameters OVERLAP_F and OVERLAP_R in the core code.

The error rate between adapter and read is set to 0.1 (default value). Processed files with less than THRESHOLD sequences are deleted. Processed files are gathered in a tar file named OUTPUT_FILE.preprocessed.tar.gz.

```
########################
# Known problems and caveats
#  -
#
########################
# Developer's todo list
#  -
#
####################################@
# FUNCTIONS
usage(){
		echo -e "$SCRIPT_NAME script:"
		echo -e "\t\tPreprocess of Illumina 2 steps PCR amplicon data.\n\t\tMerging, primer removal and filtering on seq number."
		echo -e "\t\tPlease read full information in the script first part.\n"
		echo -e "$SCRIPT_NAME usage:"
		echo -e "\t\t$SCRIPT_NAME <parameter file>"
	echo -e "\t\t(parameter file must be in the bin directory)\n"
		echo -e "\t\t#1 parameter = input directory where the fastq.gz files are stored"
		echo -e "\t\t#2 parameter = output file name without extension"
```

```
	echo -e "\t\t#3 parameter = forward target-specific primer"
	echo -e "\t\t#4 parameter = reverse target-specific primer"
	echo -e "\t\t#5 parameter = minimum length of expected overlap in flash"
	echo -e "\t\t#6 parameter = maximum length of expected overlap in flash"
	echo -e "\t\t#7 parameter = minimum number of sequences to keep the file"
}
#
######################################
# SCRIPT PARAMETERS
SCRIPT_NAME=$(basename "$0")
PARAMETER_FILE="$1"
#
######################################
# PARAMETERS TESTS
chmod +x "$PARAMETER_FILE"
dos2unix "$PARAMETER_FILE" 2>/dev/null
source "$PARAMETER_FILE"
#
# testing the first parameter : the directory
[ -e "$DIR" ] || { (usage ; echo -e "ERROR : directory $DIR does not exist!";) >&2; exit 2;}
[ -r "$DIR" ] || { (usage ; echo -e "ERROR : read permission error in directory $DIR\n";) >&2; exit 2;}
[ -w "$DIR" ] || { (usage ; echo -e "ERROR : write permission error in directory $DIR\n";) >&2; exit 2;}
#
# testing the second parameter : the output file name
[ -n "$OUTPUT_FILE" ] || { (usage ; echo -e "ERROR : output file name not informed \n";) >&2 ; exit 2;}
#
# testing the 3rd and 4th parameter : primer sequences
[ -z "$(echo "$PRIMER_F" | grep [^ATGCUatgcuNnYyRrSsWwKkMmBbDdHhVv])" ] || { (usage ; echo -e
"ERROR : #3 parameter should be a DNA sequence (degenerated bases tolerated), '$PRIMER_F' provided \n";)
>&2; exit 2;}
[ -z "$(echo "$PRIMER_R" | grep [^ATGCUatgcuNnYyRrSsWwKkMmBbDdHhVv])" ] || { (usage ; echo -e
"ERROR : #4 parameter should be a DNA sequence (degenerated bases tolerated), '$PRIMER_R' provided \n";)
>&2; exit 2;}
#
# testing the 5th and 6th  parameter : overlaps for flash (positive numbers)
echo "$MIN_FLASH" | grep -w -e "^[1-9]+$" && { (usage ; echo -e "ERROR : #5 parameter should be a
positive integer, '$MIN_FLASH' provided \n";) >&2; exit 2;}
echo "$MAX_FLASH" | grep -w -e "^[1-9]+$" && { (usage ; echo -e "ERROR : #6 parameter should be a
positive integer, '$MAN_FLASH' provided \n";) >&2; exit 2;}
#
# testing the 7th parameter : the threshold (positive or null number)
echo "$THRESHOLD" | grep -w -e "^[0-9]+$" && { (usage ; echo -e "ERROR : #7 parameter should be a null
or positive integer, '$THRESHOLD' provided \n";) >&2; exit 2;}
#

######################################
# SCRIPT VARIABLES
PRIMER_R_rev=$(echo "$PRIMER_R" | tr "[ATGCUatgcuNnYyRrSsWwKkMmBbDdHhVv]"
"[TACGAtacgaNnRrYySsWwMmKkVvHhDdBb]" | rev)
let "OVERLAP_F=$(echo "$PRIMER_F" | tr -d '[:space:]' | wc -m | tr -d '[:space:]')-1"
let "OVERLAP_R=$(echo "$PRIMER_R" | tr -d '[:space:]' | wc -m | tr -d '[:space:]')-1"
#
######################################
```

```
# CORE CODE
cd ${DIR}
log=$OUTPUT_FILE".general.log"
[ -e "$log" ] && rm "$log"
usage > "$log"
echo -e "\n$SCRIPT_NAME parameters:" | tee -a "$log"
echo -e "\t\t#parameter file:\t$PARAMETER_FILE" | tee -a "$log"
echo -e "\t\t#input directory:\t$DIR" | tee -a "$log"
echo -e "\t\t#output file name:\t$OUTPUT_FILE" | tee -a "$log"
echo -e "\t\t#forward target-specific primer:\t$PRIMER_F" | tee -a "$log"
echo -e "\t\t#reverse target-specific primer:\t$PRIMER_R" | tee -a "$log"
echo -e "\t\t#minimum length of expected overlap:\t$MIN_FLASH" | tee -a "$log"
echo -e "\t\t#maximum length of expected overlap:\t$MAX_FLASH" | tee -a "$log"
echo -e "\t\t#minimum number of sequences to keep the file:\t$THRESHOLD" | tee -a "$log"
echo -e "\n$(ls *R1_001.fastq.gz | wc -l) files to analyse:" | tee -a "$log"
#
for i in *R1_001.fastq.gz
do
#
    r1="$(basename $i)"
    #read1 file name
    r2="$(echo $r1|/bin/sed 's/R1/R2/g')"
    #read2 file name
    s="$(echo $r1|/bin/sed 's/_.*//')"
    #core sample name
    echo -e "\t\t#processing file $s"
    f=$s".flash.fastq.gz"
    #flash output file name
    c3=$s".cut3.flash.fastq"
    #cutadapt 3adapter output file name
    c35=$s".cut35.flash.fastq"
    #cutadapt 5adapter output file name
    e=$s".log.txt"
    #error file name
    [ -e "$e" ] && rm "$e"
#
    echo -e "#1 analysis = flash analysis:\n" > "$e"
    [ -s "$r1" ] && /home/bin/FLASh/1.2.11/x64/flash --threads 4 --min-overlap "$MIN_FLASH" --max-overlap "$MAX_FLASH" --max-mismatch-density 0.1 --compress "$r1" "$r2" --to-stdout > "$f" 2>> "$e" || { echo -e "$s is empty\n" >> "$e";}
    [ -s "$f" ] && (echo -e "\n#2 analysis = cutadapt analysis with reverse primer:\n" >> "$e";/home/bin/Cutadapt/1.9.1/x64/bin/cutadapt -a "$PRIMER_R_rev" "$f" --error-rate 0.1 --discard-untrimmed --match-read-wildcards --overlap "$OVERLAP_R" > "$c3" 2>> "$e";)
    [ -e "$f" ] && rm "$f"
    [ -s "$c3" ] && (echo -e "\n#3 analysis = cutadapt analysis with forward primer:\n" >> "$e";/home/bin/Cutadapt/1.9.1/x64/bin/cutadapt -g "$PRIMER_F" "$c3" --error-rate 0.1 --discard-untrimmed --match-read-wildcards --overlap "$OVERLAP_F" > "$c35" 2>> "$e" && gzip -f "$c35";)
    [ -e "$c3" ] && rm "$c3"
    [ "$THRESHOLD" -ne 0 -a -e "$c35.gz" ] && { echo -e "#4 analysis = removal of denoised files with less than $THRESHOLD sequences:\n" >> "$e"; a=$(echo "$(zcat "$c35.gz" | wc -l)/4" | bc); [ "$a" -lt "$THRESHOLD" ] && { echo -e "file removed: $a sequences only\n" >> "$e" ;rm "$c35.gz";} || { echo -e "file kept: $a sequences\n" >> "$e";};}
```

```
    [ "$THRESHOLD" -eq 0 -a -s "$c35.gz" ] && { echo -e "\n#4 analysis = removal of denoised files with less than $THRESHOLD sequences:\n\nNA" >> "$e"; }
done
[ -e $OUTPUT_FILE.preprocess.gz.tar ] && rm $OUTPUT_FILE.preprocess.gz.tar
nf=$(ls *cut35.flash.fastq.gz 2>/dev/null | wc -l)
echo -e "\n$nf files passed all steps with success!\n" | tee -a "$log"
[ "$nf" -eq 0 ] || (echo -e "list of files processed with success:" >> "$log"; tar -cvf $OUTPUT_FILE".preprocess.gz.tar" *cut35.flash.fastq.gz >> "$log";chmod a+r $OUTPUT_FILE".preprocess.gz.tar";rm *cut35.flash.fastq.gz;)
tar -cvf $OUTPUT_FILE".preprocess.log.tar" *log.txt >/dev/null; rm *log.txt #use tar -xf if you want to check a log file
#
######################################
# END
exit 0
```

File S2. Parameter file for bash script set for the minibarcode from Gillet et al. (2015).

```
#######################################
# BEFORE FIRST USE, the paths of the cutadapt and flash programs need to be appropriately set up lines 158, 159 and 161 of the Bash file
########################################
# SCRIPT PARAMETERS
DIR="/home/chapuism/coi1/" #input directory where the fastq.gz files are stored
OUTPUT_FILE="coi1run1" #output file name without extension
PRIMER_F="ATTCHACDAAYCAYAARGAYATYGG" # forward target-specific primer
PRIMER_R="ACTATAAAARAAAYTATDAYAAADGCRTG" # reverse target-specific primer
MIN_FLASH=10 # minimum length of expected overlap (to contig reads)
MAX_FLASH=300 # maximum length of expected overlap (to contig reads)
# example 1 => in a case of a amplicon length<read length (e.g. minibarcode): expected amplicon length + or - x bases of variation
# example 2 => in a case of a amplicon length>read length and of a run of high quality: 2 * expected read length - expected amplicon length + or - x bases of variation
# example 3 => in a case of a amplicon length>read length and of a run of bad quality: minimum acceptable (ex: 10) for MIN and maximum possible (length of reads; ex: 300) for MAX
THRESHOLD=0 # an entire value - the files with lower number of sequences (contigs) than the threshold will be put aside in a internal folder named 'neg'
#######################################
# Details of the library construction (useful to understand parametrization)
# Minibarcode from Gillet et al. (2015) Mammalian Biology 80: 505–509
# Target length=133b
# Forward target-specific primer=EPT-MG=ATTCHACDAAYCAYAARGAYATYGG
# Reverse target-specific primer=LepF1=ACTATAAAARAAAYTATDAYAAADGCRTG
# Construction = Adapter Flowcell + Index + Sequencing Primer + Spacer + Target-specific Forward Primer + Target + Target-specific Reverse Primer + Spacer + Sequencing Primer + Index + Adapter Flowcell
# PCR1 = Sequencing Primer + Spacer + Target-specific Primer
# PCR2 = Adapter Flowcell + Index + Sequencing Primer
# After demultiplexing:
# Read1 = Spacer + Target-specific Forward Primer + Target + Target-specific Reverse Primer + Spacer
# Read2 = Spacer + Target-specific Reverse Primer + Target + Target-specific Forward Primer + Spacer
# From 0 à 5 bases + 25 bases for LepF1 + 133 bases of target + 29 bases for EPT-MG + 0 à 5 bases (total=from 187 to 197 bases)
```
